# Multi-Level Effects of Humble Leadership on Employees’ Work Well-Being: The Roles of Psychological Safety and Error Management Climate

**DOI:** 10.3389/fpsyg.2020.571840

**Published:** 2020-11-11

**Authors:** Zheng Zhang, Peng Song

**Affiliations:** ^1^School of Business Administration, Shanxi University of Finance and Economics, Taiyuan, China; ^2^College of Business and Economics, University of Australian National, Canberra, ACT, Australia

**Keywords:** humble leadership, work well-being, psychology safety, error management climate, multi-level effect

## Abstract

Employees’ work well-being (WWB) is vital to employees’ performance and organizations’ sustainable development. This study aims to explore the role of psychological safety and error management climate (EMC) between humble leadership and WWB in Chinese organizations. Drawing upon social information processing theory, a multi-level study was conducted to test the underlying mechanisms between humble leadership and employees’ WWB. A time-lagged data of 221 team members was collected from 12 small and medium sized companies in China. Results showed that team-level humble leadership was positively related to WWB. Psychological safety and EMC both played a partial mediating role linking humble leadership and WWB. EMC positively moderated the relationship between humble leadership and psychological safety. This paper contributes to revealing the multi-level effects of humble leadership on work well-being. These findings also provide some important implications for managerial practices.

## Introduction

With the rise of positive psychology and psychology of sustainability (Di, 2017), work well-being (WWB), a form of well-being, has attracted much attention from academia (Liu et al., 2009). WWB can bring a variety of positive outcomes to employees and organizations, such as higher productivity, performance, and innovativeness (Lin et al., 2014; Honkaniemi et al., 2015; Kowalski and Loretto, 2017). Therefore, exploring different ways to improve employees’ WWB is of great significance (Choi et al., 2017).

Previous research has found that WWB is influenced by individual differences and context factors, such as positive emotions (Siu et al., 2015), work-related stress (Siu et al., 2005), high-performance work systems (Zhang et al., 2013), and organizational justice (Moliner et al., 2008). Throughout the research on the predictors of WWB, leadership has been identified as a key influential factor (Choi et al., 2017; Zhong et al., 2020). Studies have explored the influence of ethical leadership (Chughtai et al., 2015), transformational leadership (Liu et al., 2009), and authentic leadership (Rahimnia and Sharifirad, 2015) on WWB. However, we still know little about whether team-level humble leadership affects WWB, especially in the Chinese context (He et al., 2019). Humble leadership as a bottom-up leadership style, may provide more emotional and psychological resources with employees, through appreciating followers’ strengths and contributions, and building high quality relationship with them (Zhang and Liu, 2019), which increase the likelihood for employees to gain WWB. Because prior most studies on humble leadership and WWB were based on a single level (Zhong et al., 2020), it may hinder the accumulation of knowledge about the cross-level influence of team humble leadership on WWB and is not conducive to our systematic understanding the impact of group-level phenomenon (i.e., humble leadership) on WWB. Additionally, if we only suppose humble leadership at the individual level, it may indicate that all employees are independent individuals. In doing so, it ignores the effect of group-level characteristics on dependent variables (Rousseau, 1985), which in turn, fails to reveal the complex formation mechanism of WWB and reduces the external validity of the research results (Zhang, 2010). Indeed, employees are nested within a particular work group or team (Zhang, 2010), their WWB can be impacted by a high-level construct (i.e., humble leadership). A more appropriate analytical method is to consider employees as groups of followers in relation to team leaders in a multilevel approach (Chen et al., 2017). Hence, the current study makes a theoretical contribution by examining the cross-level effect of humble leadership on WWB in Chinese societies.

Although a link exists between humble leadership and WWB (Nielsen and Marrone, 2018; Zhong et al., 2020), little is known on the multi-level mechanisms of this relationship, which yields an incomplete picture of how humble leadership influences WWB. Exploring the influence of humble leadership on employee affect is important for gaining a more holistic theoretical understanding of the impact of humble leadership on employees (Wang L. et al., 2018). Meanwhile, our study also responds to the call for more research to explore the mediating mechanisms between humble leadership and WWB (Zhong et al., 2020). Thus, the second objective of our study is to uncover the underlying process through which humble leadership can affect WWB, based on social information processing (SIP) theory (Salancik and Pfeffer, 1978). Humble leadership, as a vital source of social information (Zhong et al., 2020), may send some positive signals by encouraging leader-member role reversal and legitimating uncertainty, which helps employees to eliminate interpersonal risk (Hu et al., 2018). Psychological safety can capture the individual perception of interpersonal risk (Edmondson, 1999), which in turn, influences individuals’ positive work attitude or well-being (Sharifirad, 2013; Newman et al., 2017). Additionally, Owens and Hekman (2012) proposed follower perceptions as a mechanism–legitimizing followers’ developmental journal and legitimizing uncertainty, which align with psychological safety (Walters and Diab, 2016). Thus, we propose that psychological safety will be the mediating variable that links humble leadership to WWB.

Furthermore, humble leaders are willing to acknowledge their limitations, faults, and mistakes, which fostered a perception that making mistakes is a normal and even a beneficial part of learning (Owens and Hekman, 2012). We further propose team error management climate (EMC)–a sharing perception about communicating errors, sharing and learning error knowledge, and helping in faulty situations (Van Dyck et al., 2005)–can capture the above perception in a team context. That is, humble leadership may influence employees’ well-being through EMC. As SIP theory posits, leaders act as vital sources of social information, which can shape employees’ perceptions, attitudes and behaviors (Salancik and Pfeffer, 1978). EMC is regarded as team members’ shared belief in making mistakes is safe, after they receive humble leaders who send messages of being open and teachable (Liu et al., 2017). Schein (1985) pointed out that leaders make great efforts to integrate their values and beliefs into team members’ shared understanding, which in turn, affects their attitudes. Previous research also indicated that team leadership may influence employee outcomes through team climate (Walumbwa et al., 2008; Walumbwa et al., 2010). Thus, we also examine the mediating role of EMC in the humble leadership and WWB linkage.

However, Owens and Hekman (2012) indicated that humble leadership may not always be effective, it may need supportive or resourceful environments to flourish. Following this idea and based on existing psychological safety literature (Cigularov et al., 2010; Hu et al., 2018), we propose that EMC is a possible moderator to explain the effects of humble leadership on psychological safety. Team with a strong EMC do not punish employees who make errors, but instead try to understand their errors, help them deal with the errors, and learn from these errors (Van Dyck et al., 2005; Guchait et al., 2018). In such teams, employees may have low interpersonal risk when they make errors. To the best of our knowledge, no study has examined the moderating role of EMC on the abovementioned relationships. Thus, by identifying the boundary condition of humble leadership on psychological safety, this study can contribute to the further understanding of the effectiveness of humble leadership. It is worth noting that EMC may have a direct effect on psychological safety. However, here we only explore the moderating role of EMC on the relationship of humble leadership and psychological safety, in order to examine the indirect role of EMC on psychological safety and expand the theorizing toward the moderating role.

In summary, this study is designed to examine the relationship between team-level humble leadership and WWB and the roles of psychological safety and EMC in this relationship. This study provides important implications for Chinese team management.

## Literature Review and Hypothesis Development

### Employees’ WWB

Well-being is a broad concept, and the academic circle has not reached a consensus on its definition (Zheng et al., 2015). There are two major philosophical views about well-being: one is hedonism, which defines well-being as the subjective experience of happiness; the other is eudemonism, which considers well-being as the result of individual self-actualization (Ryan and Deci, 2001). Diener (1984) followed the hedonism orientation and proposed the subjective well-being (SWB), while psychological well-being (PWB) was proposed by Ryff and Singer (2008) who inherited the eudemonism orientation. Later, some scholars took an integrative perspective to examine well-being by combining SWB and PWB, but other researchers questioned the utility of the distinction in empirical work because of their strong correlation (Fisher, 2010). In summary, different philosophical basis leads to conceptual model and research paradigms, which in turn, result in the complexity and ambiguity of employees’ well-being construct (Liu et al., 2009; Sharifirad, 2013). At present, researchers defined and operationalized the construct of employees’ well-being for their own research needs and purposes (Zheng et al., 2015). WWB is a context-specific well-being, and its definition also varies greatly, but using a multiple-measure approach to measure WWB is a trend (Zheng et al., 2015). For example, Warr (1987) has categorized concepts such as job satisfaction, job-related tension, and job-related depression as WWB. Liu et al. (2009) and Sharifirad (2013) have both proposed that WWB comprises job satisfaction, perceived work stress, and stress symptoms. Fisher (2010) argued that although a number of constructs were used to reflect some form of WWB, these constructs have in common that all refer to pleasant judgments and pleasant experiences at work. Recently, Zheng et al. (2015) also proposed that WWB is composed of pleasant judgments (i.e., job satisfaction) and pleasant experiences (i.e., positive emotions) after they reviewed employees’ well-being literature and interviewed Chinese samples. Although WWB is positively related to job satisfaction and positive affect, WWB still has a good discriminant validity (see Zheng et al., 2015). Following Fisher (2010) and Zheng et al. (2015), in present study, we define WWB as employees’ positive judgments and affect about work.

### Humble Leadership and WWB

Humility has a profound historical root. Some of the earliest writings regarding this topic come from Greek tradition and Chinese Taoism tradition (Morris, 2005). Researchers have made rapid progress in humility studies in recent 10 years, especially in the field of humble leadership. Humble leadership mainly includes two perspectives: trait view and behavior view, the former underlined leader humility is a rare personality trait. However, with the practice turns of leadership research, many scholars have begun to define humble leadership from the perspective of behavior (Owens and Hekman, 2012). Humble leadership is defined as a leadership style that leaders who admit their own mistakes and limitations, highlight followers’ strengths and contributions, and model teachability (Owens and Hekman, 2012). As a bottom-up leadership, humble leadership has some differences with similar constructs, such as authentic leadership and inclusive leadership. Both authentic leadership and humble leadership emphasize expressing yourself genuinely, but the former is subordinate-orientation, the latter is dual-orientation, that is, humble leaders improve themselves by learning while focusing on the development of employees (Ladkin and Taylor, 2010; Owens and Hekman, 2012). Furthermore, inclusive leadership and humble leadership both underline keeping openness to others opinions, appreciating employees’ strengths and contributions, and maintaining high quality exchange relationship (Nembhard and Edmondson, 2006; Owens and Hekman, 2012), but humble leaders encourage leader-member role reversal, learn modestly and give psychological freedom to employees (Owens and Hekman, 2012), which are different from inclusive leaders. Previous studies have demonstrated that humble leadership has a positive impact on employees’ attitudes or emotions, such as job satisfaction, engagement (Nielsen et al., 2010), relational energy (Wang L. et al., 2018), and psychological empowerment (Jeung and Yoon, 2016).

We propose that humble leadership may positively affect employees’ WWB. First, humble leaders exhibit clear self-consciousness, seek to be taught by employees, and appreciate followers’ strengths and contributions; and thus, employees may perceive these social cues as initiating a leader–employee role reversal (Wang L. et al., 2018), which can enhance employees’ perceived trust and support from leaders (Owens and Hekman, 2012). Research has shown that when subordinates think their leaders are trustworthy, they will have positive cognition evaluation of their work (Dirks and Ferrin, 2001). Second, humble leaders appreciate employees’ strengths and contributions publicly and legitimize and facilitate their growth and improvement, which can provide positive psychological benefits and freedom and enhance employees’ WWB (Fritz et al., 2011). Finally, humble leadership has a relational identity orientation (Nielsen et al., 2010), which makes it easier to develop a good LMX with employees (Owens and Hekman, 2012). When employees have high LMX with their leaders, they could obtain more resources, information, and empowerment (Liden et al., 1997), which in turn, lead to positive attitudes and effects, such as WWB (Sparr and Sonnentag, 2008). Recent study indicated that humble leadership is positively related to WWB (Zhong et al., 2020). Therefore, we propose:

**Hypothesis 1:** Humble leadership is positively related to WWB.

### Mediating Role of Psychological Safety

Psychological safety refers to individuals’ perceptions of the consequences of interpersonal risks in their work environment (Edmondson, 1999). The higher the individuals’ psychological safety, the lesser they have to fear the negative consequences of their own image, status, and career when they show and employ themselves freely (Kahn, 1990). Psychological safety often been conceptualized as a team-level construct (e.g., Edmondson, 1999). However, recent research has begun to explore psychological safety at the individual and organizational levels (Newman et al., 2017). In this study, we focus on the individual level of psychological safety, in order to unveil the individual psychological mechanism of team level of humble leadership on WWB.

We argue that humble leadership has a positive effect on psychological safety. First, from the perspective of SIP theory, humble leadership is an important social cue, which may send positive social information to employees (Wang L. et al., 2018). For example, humble leaders who exhibit openness to new ideas, have a habit of listening before speaking, and appreciate others’ contributions (Owens and Hekman, 2012; Randel et al., 2017) may convey the information that leaders are inclusive (Carmeli et al., 2010). When perceived by employees, such information will help improve their psychological safety (Nembhard and Edmondson, 2006; Carmeli et al., 2010). Importantly, humble leaders encourage employees to keep trying and perceive mistakes as tools for growth and take responsibility for employees’ mistakes (Owens and Hekman, 2012). These cues send signals to employees that making mistakes and taking risks are acceptable (Hu et al., 2018). Thus, employees are likely to show themselves freely or take risks without fear of adverse influences. Second, with the role modeling of humble leaders, team members will also imitate their leaders when it comes to showing their own shortcomings, appreciating the advantages and contributions of others, and learning modestly (Bandura, 1977), which promotes the formation of collective humility in the team (Owens et al., 2013; Rego et al., 2017). Moreover, the higher the collective humility, the higher quality of interpersonal relationships will develop among team members (Peters et al., 2011). Carmeli et al. (2008) have suggested that high interpersonal relationships are positively related to psychological safety. Finally, Edmondson (2004) has noted that three types of leaders’ behaviors are the antecedents of psychological safety. These behaviors include accessibility, continuous invitation of input, and modeling openness and fallibility, all of which can be shown by humble leaders (Kayla and Walters, 2016). Empirical studies have shown that humble leadership can increase employees’ psychological safety (Kayla and Walters, 2016).

We further argue that psychological safety is positively related to WWB. Although direct empirical evidence supporting the relationship between psychological safety and WWB is scarce, indirect evidence can be found in some theories and literature. For example, employees with a high level of psychological safety appear to experience a great sense of freedom, support, and respect at their work (Chen et al., 2014). According to social exchange theory (Blau, 1964), employees are likely to reciprocate their leaders’ or organizations’ good intentions with positive attitudes or affection. Moreover, from the perspective of conservation of resources theory (Hobfoll, 1989), individuals are willing to make resource investment when the external threat is low. Thus, individuals are likely to invest their resources (i.e., WWB) to their works when their psychological safety is high (Newman et al., 2017). Sharifirad (2013) has pointed out that psychological safety is positively related to employees’ well-being (job satisfaction, work-related stress, and stress symptoms). Chen et al. (2014) have indicated that a significantly positive relationship exists between psychological safety and affective commitment, which is a dimension of WWB (Heffernan and Dundon, 2016). Accordingly, the higher the psychological safety of employees, the greater the positive attitudes and affection they exhibit (Kirk-Brown and Van Dijk, 2016).

According to SIP theory (Salancik and Pfeffer, 1978), leadership behavior is a social information, which influences individuals’ subsequent attitudes and behaviors by affecting their psychological perception. Relevant study has shown that transformational leadership has a positive impact on employees’ WWB through psychological safety (Sharifirad, 2013). We propose that humble leadership has a positive effect on employees’ WWB through psychological safety. Thus, we propose:

**Hypothesis 2:** Psychological safety mediates the relationship between humble leadership and WWB.

### Mediating Role of EMC

Error management climate refers to “shared practices and beliefs related to communicating errors, sharing error knowledge, helping in faulty situations, and quickly detecting and handling errors” (Van Dyck et al., 2005, p. 1229). In this study, we focus on team level EMC, and argue that EMC may mediate the relationship between humble leadership and WWB.

Social information processing theory suggests that leader behavior as a vital social information cue is helpful to shape employees’ perceptions about the work environment, and in turn, affects individuals’ attitudes and behaviors (Salancik and Pfeffer, 1978). Many studies pointed that leaders as climate engineers and they play an important role in the development of climate (Naumann and Bennett, 2000; Walumbwa et al., 2010), and the corresponding climate may mediate group-level leader behavior on employees’ outcomes (Walumbwa et al., 2008). For example, Walumbwa et al. (2008) found that procedural justice climate mediated the relationship between contingent reward leader behavior and employee satisfaction with supervisor, organizational commitment, and OCB. Whereafter, Walumbwa et al. (2010) identified that procedural justice climate and service climate mediated the link between servant leadership and OCB. Thus, based on SIP theory and team climate literature, we propose humble leadership may influence WWB through fostering a specific work team climate (i.e., error management climate). We first argue that humble leadership is positively related to EMC. Humble leaders acknowledge personal faults and mistakes, and encourage team members to learn from mistakes. These may send some signals that making a mistake is normal, and learning and growing from mistakes is more important than blaming (Owens and Hekman, 2012). For this reason, we speculate that humble leadership may positively related to EMC. In addition, leaders’ social modeling will impact employees’ attitudes toward errors and trigger employees to imitate their leaders (Rego et al., 2017). Employees are more likely to communicate their mistakes, share error knowledge and express more inclusiveness when others make mistakes (Zhong et al., 2020), which can help to create a climate of error management. Furthermore, humble leaders give more psychological freedom to employees and take responsibility for their team (Owens and Hekman, 2012), employees will feel encouraged and supported by their leaders, and they will do more trial and error without worrying about failure (Owens and Hekman, 2012), thus, it can help to shape a good EMC.

We further argue that EMC is positively related to WWB. A team with a high level of EMC encourages team members to communicate, help and learn from each other after error happens (Maurer et al., 2017), it may help employees to perceive coworker support (Casey and Krauss, 2013). Having support from coworkers can influence employees’ attitude and positive feelings toward their jobs (Karatepe, 2012), and lessen emotional exhaustion (Zhong et al., 2020). Additionally, in a high level of EMC, team sees errors as a natural phenomenon and an opportunity to learn (Keith and Frese, 2005), team members are more willing to discuss errors, which help to reduce stress and improve positive emotion. In contrast, employees are likely to be anxious about being punished for their mistakes in the team with high EMC, this may induce job stress and fatigue, which in turn influence WWB negatively (Lu et al., 2011). As such, we propose:

**Hypothesis 3:** EMC mediates the relationship between humble leadership and WWB.

### Moderating Role of EMC

Researchers have suggested that organizational climate can provide a context in which individuals evaluate workplace safety (Mearns and Flin, 1999; Cigularov et al., 2010). In addition, Owens et al. (2013) have noted that context factors could influence the effectiveness of humble leadership.

We argue that EMC moderates the relationship between humble leadership and psychological safety. If a team has a high level of EMC, then team members consider errors as a common phenomenon (Keith and Frese, 2005). EMC emphasizes employees’ learning, communication, and development as a result of errors (Maurer et al., 2017), and encourages members to voice their views, discuss mistakes openly, and learn from their errors (Rupert et al., 2019). Therefore, team members do not fear being blamed for making mistakes. Humble leaders legitimize employees’ processing of development by telling them that making mistakes is a normal and a beneficial part of learning (Owens and Hekman, 2012). EMC is consistent with the information conveyed by humble leaders. Thus, EMC can provide a context that strengthens the influence of humble leaders, which in turn, enhances employees’ psychological safety. By contrast, if a team has a low level of EMC, team members view errors negatively and emphasize punishing and blaming errors (Van Dyck et al., 2005). Employees will not likely show themselves freely in such a climate, although team leaders take responsibility for errors. Thus, if a team lacks a shared belief in error management, humble leaders are less likely to provide employees with psychological safety. Additionally, Wang X. et al. (2018) have pointed out that employees tend to believe that their leaders are trustworthy and do good in exchange under a high level of EMC. Thus, employees may treat humble leadership positively under high EMC, which in turn, improves the positive influence of humble leadership on psychological safety. Accordingly, we propose:

**Hypothesis 4:** EMC positively moderates the relationship between humble leadership and psychological safety. That is, the higher the EMC, the stronger the positive relationship between humble leadership and psychological safety.

### Moderated Mediation Effect

Combined Hypothesis 2 and Hypothesis 4, we propose a moderated mediation model, that is, the mediating role of psychological safety between humble leadership and WWB will be moderated by EMC. When EMC is higher, humble leadership has a stronger effect on employees’ psychological safety, and the improvement of psychological safety is likely to increase their perception of freedom, support, and respect at their work (Chen et al., 2014), which in turn, enhancing WWB. In contrast, when EMC is lower, it may weaken the stimulating effect of humble leadership on employees’ psychological safety, and the lower level of psychological safety may lead to passive attitudes and emotions toward work (Nixon et al., 2015; Kirk-Brown and Van Dijk, 2016), which ultimately weakened the WWB of employees. Thus, we propose:

**Hypothesis 5:** EMC moderates the indirect effect of humble leadership on WWB through psychological safety. That is, with the improvement of EMC, the mediating role of psychological safety between humble leadership and WWB becomes stronger.

**FIGURE 1:**
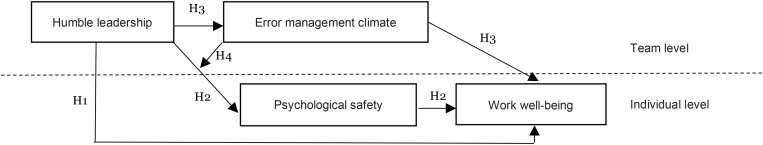
Theoretical model of the present study.

The hypothesized model is presented in Figure 1.

## Methods

### Procedure and Samples

The research data, adopting the field survey at two time points, were collected from 12 small and medium sized companies (the number of employees is less than 200) located in Taiyuan, Zhengzhou, and Beijing, China. Among these companies, six were in the service industry, four were in the manufacturing industry and two were in the education industry. We communicated fully with the company leaders in advance and coded the participants after the leaders’ agreement. Before sending out the questionnaires, we introduced the purpose of our study to the participants and emphasized that this survey was purely academic and fully anonymous and voluntary. At time 1, participants were asked to assess humble leadership, psychological safety, EMC, and demographic variables. Two months later, they reported their WWB at time 2. After the two-wave questionnaires were matched, and teams with less than 3 members were deleted, 221 valid samples of 67 teams were finally obtained after eliminating the invalid questionnaires, the effective recovery rate is 84.5%. Each team leader rated 3.3 employees on average. Among the 221 employees, 51.1% were male, 54.3% of participants had a bachelor’s degree, and 33.9% had a junior college degree. The average age was 30.81 years (*SD* = 0.51). The job nature of these employees is mainly marketing, technology, and HR. Among the 67 leaders, 69.8% were male, 13.7% had a master degree, 50.7% had a bachelor’s degree, 27.4% had a junior college degree, and the average age was 38.88 years (standard deviation = 10.11).

### Measures

All measures used a five-point Likert scale (1 = strongly disagree to 5 = strongly agree) and employee self-reported method. We translated the English scale to Chinese under a standard back-translation method (Brislin, 1970).

#### Humble Leadership

We used a nine-item scale developed by Owens et al. (2013) to measure humble leadership. A sample item was “Our team leader actively seeks feedback, even if it is critical.” Cronbach’s α was 0.87 in this study. Given that humble leadership was a team-level construct (Hu et al., 2018), we used within-group reliability (ICC1), reliability of group mean (ICC2), and within-group agree indices (rwg) to justify whether aggregating individual members’ rating to the team level was appropriate. ICC1, ICC2, and rwg were 0.24, 0.52, and 0.91, respectively. These three indicators were well above the acceptable values of 0.12, 0.47, and 0.70 (James, 1982), supporting aggregation.

#### Psychological Safety

We used a seven-item scale developed by Edmondson (1999) to access psychological safety. A sample item was “Taking risks in this team is safe.” Cronbach’s α was 0.75 in this study.

#### Error Management Climate (EMC)

To measure team-level EMC, we used a 16-item scale adopted from Cigularov et al. (2010), which includes learning form errors (four items), thinking about errors (five items), error competence (three items), and error communication (four items). Sample items were “When mastering a task, our team can learn a lot from their mistakes” and “When someone makes an error in our team, he/she shares it with others to prevent the occurrence of the same mistake.” Cronbach’s α was 0.87 in this study. ICC1 and rwg of EMC were 0.15 and 0.96, exceeding the cut-off values of 0.12 and 0.70, respectively. However, ICC2 was 0.36, which was below the acceptable value of 0.47. This result may be due to fewer team members (i.e., the average team size is less than eight persons) (Bliese, 2000). Chen and Bliese (2002) pointed out that if the aggregation was supported by theory and rwg and between-group variance were significant, aggregation was feasible. Based on this finding, we conducted a one-way analysis of variance (ANOVA). The result showed that a significant between-group variance exists in team-level EMC (*F* = 1.59, *p* < 0.05).

#### Work Well-Being (WWB)

Work well-being was assessed using Zheng et al. (2015) six-item scale. A sample item was “In general, I feel fairly satisfied with my present job.” Cronbach’s α was 0.84 in this study. This scale was used in many studies, such as Miao and Cao (2019) and Fu et al. (2020).

#### Control Variables

To eliminate the possible influence of other variables on WWB, we controlled for gender, age, education of employees, and teams’ size and development stage, because previous study has found these variables have an influence on WWB (Paulin and Griffin, 2016; Zhong et al., 2020). Gender may influence WWB, for female employees, they may have fewer positive job attitudes or emotions than male employees because they have difficulties in getting promoted (Clark, 1997). Age also may be positively related to WWB, that is because older employees have better supplies-values fit, they may get more positive perceptions from jobs and workplaces (Piszczek and Pimputkar, 2020). In addition, education level differences may have an impact on WWB, because compared with higher educated employees, lower educated employees may have a high physical workload and less favorable work environment (Akkermans et al., 2009). Furthermore, team size may affect employees’ WWB. Prior research indicated that as the team size becomes larger, members have difficulty in maintaining good interpersonal relationships and getting individual recognition, and their leaders are less likely to display consideration behaviors. Therefore, they may experience less satisfaction (Mullen et al., 1989). Finally, the team development stage may be related to WWB, because members’ trust and interpersonal relationship is lower in the early stages of team development (e.g., forming and storming) than the late stages (e.g., maturing and declining), there are a lot of conflicts within the team, thus, they may not get many positive emotions from work (Tuckman, 1965).

## Results

### Preliminary Analysis

Confirmatory Factor Analysis (CFA) and Common Method Variance (CMV). We used Harman’s single factor test to check for possible CMV (Podsakoff et al., 2003). As stated by this approach, CMV will be exist if a single factor explains most of the covariance of all variables. An exploratory factor analysis (EFA) results showed that the first unrotated factor explains 28.44% of the variance in our research’s variables. The variance explained by the first factor does not exceed 40%, we can conclude the CMV is not serious in our study (Ashford and Tsui, 1991). Additionally, we conducted CFA by using Mplus 7.0 to examine the distinctiveness of member-rated variables (i.e., humble leadership, psychological safety, EMC, and WWB). Considering the small sample size relative to the measurement items (Liu et al., 2013), we used random item parceling for every construct before conducting the analyses. Because random algorithm is not affected by scales and samples (Matsunaga, 2008). As shown in Table 1, the hypothesized four-factor model was a better fit to the data than other alternative models. Given that we collected data from the same employees at the same time, this study examined CMV by using the unmeasured latent method factor approach (Podsakoff et al., 2003). Specifically, we added one common factor with all the measures as indicators to the four-factor model and contrasted the fit index. If the five-factor model’s (i.e., adding the common factor) fit index improved significantly compared with the four-factor model, then CMV exists. The five-factor model’s χ^2^, CFI, TLI, and RMESA were 41.42, 0.97, 0.96, and 0.06, respectively. The χ^2^ was changed significantly (Δχ^2^ = 11.53, Δdf = 2), given that it was easily influenced by the sample size. However, other fit indices did not improve significantly (both less than 0.02) (Williams et al., 1989). Hence, CMV was not a serious problem in this study. Furthermore, we also examined the construct validity by performing a multi-level CFA analysis according to Dyer et al. (2005) five-steps procedure. Results indicated that the fit indexes of multi-level model (χ^2^ = 51.50, *df* = 48, CFI = 0.99, TLI = 0.99, RMSEA = 0.02) are better than the single-level model (χ^2^ = 29.89, *df* = 21, CFI = 0.99, TLI = 0.98, RMSEA = 0.04). Thus, our data more fits a multi-level factor structure.

**TABLE 1:** CFA results of study variables.

Models	χ^2^	df	Δχ^2^	CFI	TLI	RMSEA
Four-factor model: HL, EMC, PS, WWB	29.89	21	–	0.99	0.98	0.04
Three-factor model: HL + EMC, PS, WWB	106.39	24	76.5**	0.88	0.81	0.13
Two-factor model: HL + EMC + PS, WWB	162.37	26	132.48**	0.79	0.71	0.15
One-factor model: HL + EMC + PS + WWB	175.54	27	147.65**	0.78	0.70	0.16

**p < 0.01. HL, humble leadership; EMC, error management climate; PS, psychological safety; WWB, work well-being; “+” represents the combination of factors; CFI, comparative fit index; TLI, Tucker–Lewis index; RMSEA, Root Mean Square Error of Approximation; df, degree pf freedom; and Δχ^2^, Chi-square differences.

#### Descriptive Statistics

Table 2 shows the means, standard deviations, and correlations among measures at the individual and team levels. PS is positively related to WWB (β = 0.42, *p* < 0.01), and HL is positively related to EMC (β = 0.66, *p* < 0.01). The correlations are lower than 0.75 of the multicollinearity thresholds (Tsui et al., 1995).

**TABLE 2:** Means, standard deviations, and correlations among variables.

Variable	*M*	*SD*	1	2	3	4
**Individual-level**						
(1) Gender	1.49	0.51				
(2) Age	30.81	8.57	–0.04			
(3) Education	3.40	0.73	–0.03	0.24**		
(4) PS	3.45	0.64	0.05	0.20**	0.11	
(5) WWB	3.88	0.65	–0.06	0.25**	0.14*	0.42**
**Team-level**						
(1) Team size	3.29	0.71				
(2) Team development	2.27	0.68	0.11			
stage						
(3) HL	3.84	0.44	0.16	0.15		
(4) EMC	3.99	0.30	0.09	0.11	0.66**	

n = 221 individuals, N = 67 teams. For gender, male = 1, female = 2. For age, the measure is the individuals’ real age. For education, doctoral degree = 1, master degree = 2, bachelor degree = 3, junior college degree = 4, high school and below. Team size is the number of individual members in a team. For team development stage, forming stage = 1, norming stage = 2, maturing stage = 3, declining stage = 4. *p < 0.05, **p < 0.01.

### Hypothesis Tests

HLM6.08 was used to test the cross-level effect of this study. Following Hofmann and Gavin’s (1998) suggestions, we used grand-mean centering’s team-level and individual-level variables for analysis. First, we examined two null models, which were used to determine whether significant between-group differences exist in psychological safety and WWB at the individual level. The results showed that the between-group variance of psychological safety (τ_00_) was 0.14, within-group variance (σ^2^) was 0.27, χ^2^(70) = 194.56, and *p* < 0.001, indicating that 34.1% of the variance in psychological safety was at team level. Moreover, the between-group variance of WWB (τ_00_) was 0.13, within-group variance (σ^2^) was 0.30, χ^2^(70) = 160, and *p* < 0.001, meaning that 30.2% of the variance in WWB was at team level.

Then, we tested the main effect, namely, Hypothesis 1. Based on the null model, we added control variables (i.e., gender, age, education of employees, team size, and team development stage) and humble leadership, as shown in Model 3, Table 3. The results showed that humble leadership was positively related to WWB (γ = 0.71, *p* < 0.001). Thus, Hypothesis 1 was supported.

**TABLE 3:** HLM results for hypothesis testing.

Variable	PS			WWB					
		
	Null Model	Model 1	Model 2	Null Model	Model 3	Model 4	Model 5	Model 6	Model 7
Intercept	3.47***	3.69***	3.77***	3.88***	3.67***	3.03***	3.87***	3.63***	3.85***
**Individual-level**									
Gender		0.05	0.04		–0.06	–0.06	–0.07	–0.07	–0.07
Age		0	0		0.01	0.02*	0.01	0	0
Education		0.05	0.02		0	0.01	–0.04	–0.01	–0.03
PS						0.40***	0.39***		
**Team-level**									
Team size		−0.20*	−0.18***		–0.03	0.03	–0.03	–0.01	–0.03
Team development stage		0.03	0.05		0.09	0.11	0.10	0.12	0.11^†^
HL		0.42**	0.07		0.71***		0.69***		0.39**
EMC			0.91***					1.10***	0.77***
HL*EMC			0.62*						
Variance decomposition									
Within-group variance σ2	0.26	0.27	0.27	0.30	0.29	0.25	0.24	0.29	0.29
Between-group variance τ_00_	0.14***	0.10***	0.05**	0.13***	0.03^†^	0.12***	0.04**	0.02	0

The regression coefficients are all non-standardized coefficients under the robust standard errors. ^†^p < 0.1, *p < 0.05, **p < 0.01, ***p < 0.001. HL, humble leadership; EMC, error management climate; PS, psychological safety; and WWB, work well-being. HL*EMC denotes the interaction item for HL and EMC.

We tested Hypothesis 2 according to the following procedure suggested by Baron and Kenny (1986): (1) the independent variable (HL) was related to dependent variable (WWB), as supported by Hypothesis 1; (2) the independent variable (HL) was related to mediator (PS) (Model1, γ = 0.42, *p* < 0.01); (3) the mediator (PS) was related to the dependent variable (WWB) (Model4, γ = 0.40, *p* < 0.001); and (4) the effect of independent variable (HL) must be reduced or disappeared after controlling the mediator (PS). As shown in Model 5, Table 3, the significant coefficient of humble leadership’s influence on WWB decreased from 0.71 (see Model 3) to 0.69, indicating that psychological safety played a partial mediating role on the relationship between humble leadership and WWB. Thus, Hypothesis 2 was supported.

Likewise, we test Hypothesis 3 by adopting the above procedure: (1) the independent variable (HL) was related to dependent variable (WWB), as supported by Hypothesis 1; (2) the independent variable (HL) was related to mediator (EMC) (γ = 0.66, *p* < 0.01); (3) the mediator (EMC) was related to the dependent variable (WWB) (Model6, γ = 1.10, *p* < 0.001); and (4) the effect of independent (HL) must be reduced or disappeared after controlling the mediator (EMC). As shown in Model 7, Table 3, the significant coefficient of humble leadership’s influence on WWB was decreased from 0.71 (see Model 3) to 0.39, indicating that EMC played a partial mediating role on the relationship between humble leadership and WWB. Thus, Hypothesis 3 was supported.

**FIGURE 2:**
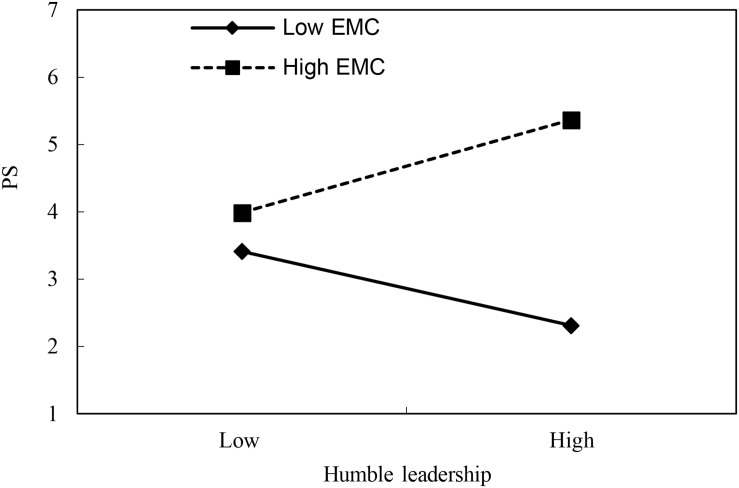
Moderating role of EMC on the relationship between humble leadership and psychological safety.

We examined Hypothesis 4 which states that EMC moderated the relationship between humble leadership and psychological safety. As shown in Model 2, Table 3, the interaction terms of humble leadership and EMC were significant and positive (γ = 0.62, *p* < 0.05). According to Aiken and West’s (1991) approach, we have plotted the relationship between humble leadership and psychological safety under the mean plus one standard deviation (*M* + *SD*) and minus one standard deviation (*M* − *SD*) of EMC to further clarify the moderating effect of team-level EMC, as shown in Figure 2. The interaction plot exhibited that the relationship between humble leadership and psychological safety was stronger (simple slope = 0.69, *t* = 6.58) when EMC was high, whereas the relationship between humble leadership and psychological safety was lower when EMC was low (simple slope = −0.55, *t* = −5.244), the slope difference is significant (Δslope = 1.24, *p* < 0.05). Therefore, Hypothesis 4 was supported.

Finally, we further tested Hypothesis 5 that EMC moderated the indirect effect of humble leadership on WWB through psychological safety by using Mplus7.0 and R4.0.2. As shown in Table 4, the results showed that when EMC was high, the indirect effect of psychological safety between humble leadership and WWB was 0.145 (95%LLCI = −0.269, ULCI = −0.047), the confidence intervals did not include 0; when EMC is low, the indirect effect of psychological safety was 0.056 (95%LLCI = −0.028, ULCI = 0.157), the confidence intervals included 0, and between-group difference was 0.088 (95%LLCI = −0.001, ULCI = 0.199), the confidence intervals included 0, it indicated that the moderated mediation did not exist, that is, the indirect effect of humble leadership on WWB was not moderated by EMC. Therefore, Hypothesis 5 was not supported.

**TABLE 4:** Moderated mediation testing.

Dependent	EMC	Effect	*SE*	Low 95%CI	High 95%CI
WWB	High	0.145	0.055	−0.269	−0.047
	Low	0.056	0.047	−0.028	0.157
	Difference	0.088	0.050	−0.001	0.199

**Bootstrapped results are based on 20,000 samples*.*

## Discussion

Based on social information processing theory and related literature, we investigated the cross-level impact of humble leadership on WWB, especially the role of psychological safety and EMC. Using a sample composed of Chinese employees, we tested our hypotheses. The results showed that team-level humble leadership was positively related to WWB, psychological safety and EMC mediated the relationship between humble leadership and WWB, and EMC moderated the relationship between humble leadership and psychological safety, but EMC did not moderate the indirect effect of psychological safety between humble leadership and WWB.

### Theoretical and Practical Implications

Our study has some theoretical contributions. First, our findings suggest that team-level humble leadership has a significantly positive relationship with WWB. As mentioned above, past research has linked humble leadership to various positive outcomes (Lin et al., 2014; Chughtai et al., 2015). However, to our knowledge, existing study has rarely explored the relationship between team-level humble leadership and WWB, especially in the Chinese context. Therefore, we explore the cross-level effects of humble leadership on WWB, which extend the previous findings (e.g., Owens et al., 2013; Nielsen and Marrone, 2018) on the relationship between humble leadership and employees’ attitudes, emotions, and behaviors, such as job satisfaction, job engagement, trust in the leader, provide evidence for the positive effect of team humble leadership on employee’s WWB in China, and further enrich the knowledge about the influence of team-level context (i.e., humble leadership) on WWB.

Second, by verifying the mediating effect of EMC and psychological safety in the linkage between humble leadership and WWB, this study can capture a more complete picture of how humble leadership influences WWB and contribute to understanding the effectiveness of humble leadership through different mechanisms. Although previous research has made much effort on clarifying the mechanisms underlying the relationship between humble leadership and team outcomes or individual outcomes through SIP theory (e.g., Rego et al., 2017; Hu et al., 2018; Zhu et al., 2019; Zhong et al., 2020), little is known about the multilevel processes between humble leadership and employees’ WWB. Following recent research, we believe that SIP theory can also provide a key to opening the “black box” of mechanism between humble leadership and WWB. Compared with other theories, SIP theory (Salancik and Pfeffer, 1978) not only unveils the mediating role of work setting, for example, social information helps individuals and teams to understand their work environment, which in turn, influences their attitudes and behaviors, but also emphasizes people’s interpretation of social information is based on certain contextual characteristics. Based on SIP theory, humble leadership as a social information may send some signals to employees or their team and further influence their positive perceptions about work or work environment, which, in turn, have an impact on WWB. Thus, we have proposed and tested that psychological safety and EMC both act as the mediators between above variables. Our results are not only consistent with the cross-level theoretical model of Walumbwa et al. (2010) proposed that “leadership—team climates/individual perceptions—employee outcomes,” but also extend the application of social information processing theory in humble leadership and WWB research field. These findings also respond to a call for more research exploring the mediating mechanisms between humble leadership and WWB (Zhong et al., 2020).

Third, we demonstrated the moderating role of EMC between humble leadership and psychological safety. As mentioned above, EMC also acted as a mediator between humble leadership and WWB. It may cause a confusing sense of the role of EMC. However, the fact that previous research has showed that team-level process variables can both act as mediator and moderator between team-level independent variables and individual-level dependent variables (Walumbwa et al., 2010). For example, Hofmann et al. (2003) argued that the specific climate within a team served to emphasize the expectations for members when they responded to leaders’ affects. Liao and Chuang (2007) also pointed out that service climate as a situational enhancer of leadership effects. Our results suggested that humble leadership as a social information fosters positive climate is more likely to strengthen the relationship between humble leadership and psychological safety. For one thing, the findings are consistent with the emphasis of social information processing theory that “people’s interpretation of social information is based on a certain social context” (Salancik and Pfeffer, 1978); for another thing, they are an important extension of the views of Hofmann et al. (2003) and Liao and Chuang (2007).

Finally, our results did not support that EMC moderates the indirect effect of psychological safety between humble leadership and WWB. A plausible explanation is that EMC affecting the mediating role of psychological safety between humble leadership and WWB is constrained by other situational factors, such as leader-member exchange differentiation (Henderson et al., 2009), which refers to the exchange relationship quality difference between team leaders and different members. Hooper and Martin (2008) showed that LMX differentiation leads to relational team conflict, which in turn, influences employees’ job satisfaction and well-being negatively. As a result, it may statistically offset the positive effect of EMC on WWB.

The present study also has important implications for managerial practices. First, considering the positive role of humble leadership, organizations should focus on cultivating and shaping the humble behaviors of team leaders. For example, such actions can be initiated by encouraging team leaders to admit their own shortcomings and limitations, appreciate the advantages of others, and learn modestly. Second, team leaders should pay much attention to the psychological safety of employees and create a good EMC. Leaders should show as much humility as possible in their work, which in turn, reduces employees’ perception of interpersonal risk and improves the level of EMC. Finally, the team should devote to creating an inclusive climate for errors, mutual trust, and mutual assistance among members to provide favorable environmental support and inspire humble leaders further, which may enhance psychological safety of employees.

### Limitations and Future Research Directions

This present study has three limitations as follows. First, although our research data was collected from a two-wave survey of Chinese employees, independent variable and mediator variable were both collected at the same time. Thus, the causal conclusion could not be inferred confidently. Additionally, 2 months is a short period and dramatic changes in mean WWB are unlikely during such a short period, so a long-time longitudinal research design is more appropriate to ascertain causal relationships. For example, we can collect the data on humble leadership and EMC from supervisors’ leaders at time1 and psychological safety and WWB from employees at time 2 and time 3, respectively, every time interval may be at least 6 months. Furthermore, our data from different types of industry and firm, although the differences among different industries or firms are not very significant by using ANOVA, other industry or firm level variables as control variables deserve to be explored in future research in order to strengthen the rigor of the study.

Second, we collected data from employees by self-reported method. Hence, our research results may be influenced by CMV. Prior extensive studies have used the self-reported approach to measure leadership and employees’ well-being (e.g., Liu et al., 2009; Chughtai et al., 2015). To reduce CMV, according to the suggestions of Podsakoff et al. (2003) and refer to some similar studies (e.g., Atwater and Carmeli, 2009), we collected data at two time points. The latent variable approach has also indicated that the method variance is not a major problem in the current study. Indeed, some research has suggested that whether to apply a self-reported method depends on the nature of the variables (Rahimnia and Sharifirad, 2015). Some variables which represent internal psychological and physiological states, such as psychological safety and WWB. The most appropriate approach to measure these variables is directly self-report from the individuals who experience them (Rahimnia and Sharifirad, 2015). Additionally, the self-report design can be very useful in providing a picture of how individuals feel about and view their work, and tell us about the intercorrelations among different perceptions and feelings (Spector, 1994). Nevertheless, to mitigate the negative impact of this problem, future research can gather data from different sources (Podsakoff et al., 2003). For example, humble leadership can be assessed by supervisors’ leaders, EMC and psychological safety are reported by employees, and WWB could be rated by employees’ coworkers or family members.

Third, SIP theory has good explanatory power for the mechanisms between leadership behavior and individual attitudes and behaviors (Salancik and Pfeffer, 1978). Our model is based on SIP theory, which helps to explain how and when humble leadership affects WWB. As mentioned above, although the linkage between humble leadership and WWB was not established by SIP theory, this theory has been used in previous humble leadership research (e.g., Liu et al., 2017; Hu et al., 2018). Thus, whether other theories, such as social exchange theory (Blau, 1964) or self-determination theory (SDT; Deci and Ryan, 1985), can provide a new insight and further construct the overall theoretical model need to be probed in future. Furthermore, although self-determination theory, which posits that three basic psychological needs are essential for well-being, may be a good framework for our study, we did not construct a theoretical model based on this theory, thus, future research can examine what best explains the relationship between humble leadership and WWB through different mediators by comparing SIP theory and SDT.

Fourth, we explore the moderating role of EMC between humble leadership and psychological safety, and the moderated mediation effect of EMC on psychological safety. However, we don’t examine the direct relationship of EMC and psychological safety in order to expand the theorizing toward the moderating role, although there is a positive relationship between them. Additionally, our results do not support that EMC moderates the indirect of psychological safety between humble leadership and WWB. Although we had tried to explain the reason that EMC may be impacted by other team context, such as leader-member exchange differentiation, further research should explore other contextual variables to improve our theoretical model.

Finally, we construct a cross-level model based on current theory limitations to link humble leadership and WWB through EMC and psychological safety. However, the correlation between humble leadership and EMC is a little high (0.66), and the standard deviation of EMC is too small (0.30), so the current data may have limited variances in the variable. To deal with these issues, we adopt multi-level CFA analyses on all variables according to Dyer and his partners’ procedure (2005), the results show that overall, the fit indexes of multi-level model are better than single-level model. But the fit index of between model is poor, it may be related to the small sample size at team-level or model complexity, thus, future research needs to expand the sample size of team-level and reduce the complexity of the model.

## Data Availability Statement

The raw data supporting the conclusions of this article will be made available by the authors, without undue reservation.

## Ethics Statement

The studies involving human participants were reviewed and approved by the School of Business Administration at Shanxi University of Finance and Economics. The participants provided their written informed consent to participate in this study.

## Author Contributions

ZZ designed the research and wrote the manuscript. PS collected and analyzed the data, and they amended the manuscript. Both authors contributed to the article and approved the submitted version.

## Conflict of Interest

The authors declare that the research was conducted in the absence of any commercial or financial relationships that could be construed as a potential conflict of interest.
